# Supplementary figures and images for: A novel acidification mechanism for greatly enhanced oxygen supply to the fish retina
Source: eLife. 2020 Aug 25;9:e58995. doi: 10.7554/eLife.58995 (PMC7447425; doi:10.7554/eLife.58995)

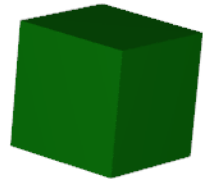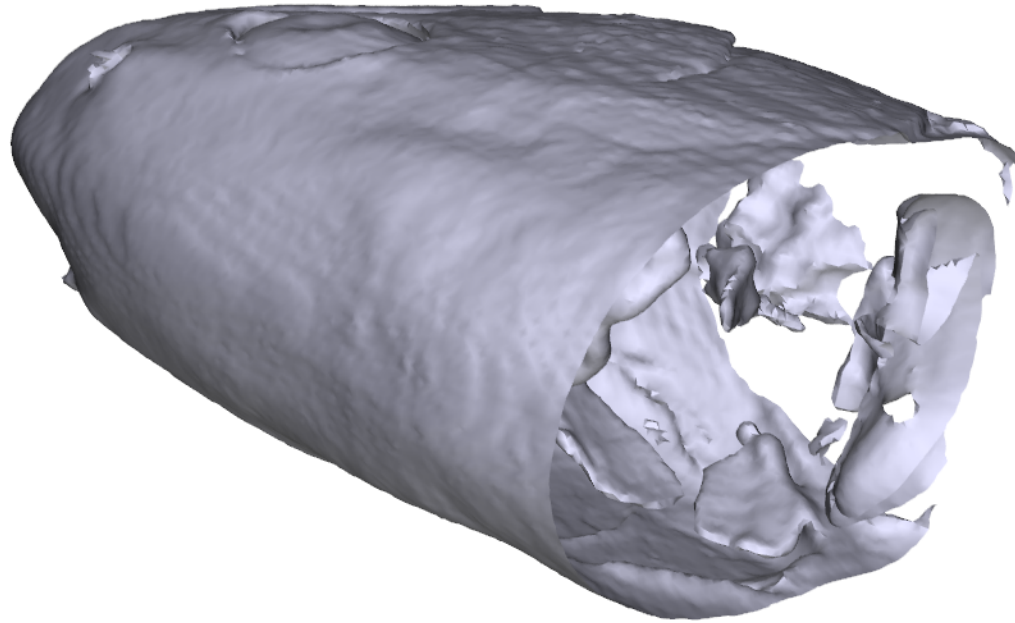

Supplement: Supplementary file 1. — Micro-CT-generated interactive overview of the ocular vasculature in a rainbow trout injected with a radiopaque contrast agent in the ventral aorta. Open the file in Adobe Acrobat Reader nine or higher and activate the 3D feature by clicking on the model. Then use the cursor to interact with the model or select pre-defined views similar to Figure 1—figure supplement 1. [file elife-58995-supp1.pdf]
